# Epileptiform Neuronal Discharges Impair Astrocyte Syncytial Isopotentiality in Acute Hippocampal Slices

**DOI:** 10.3390/brainsci10040208

**Published:** 2020-04-02

**Authors:** Qi Wang, Wei Wang, Sydney Aten, Conrad M. Kiyoshi, Yixing Du, Min Zhou

**Affiliations:** 1 Department of Neuroscience, The Ohio State University Wexner Medical Center, Columbus, OH 43210 USA; Qi.Wang@osumc.edu (Q.W.); wwang@hust.edu.cn (W.W.); Aten.19@osu.edu (S.A.); kiyoshi.1@osu.edu (C.M.K.); 2 Department of Physiology, School of Basic Medicine, Tongji Medical College, Huazhong University of Science and Technology, Wuhan 430030, China

**Keywords:** astrocytes, gap junction, astrocyte syncytial isopotentiality, epilepsy, potassium homeostasis

## Abstract

Astrocyte syncytial isopotentiality is a physiological mechanism resulting from a strong electrical coupling among astrocytes. We have previously shown that syncytial isopotentiality exists as a system-wide feature that coordinates astrocytes into a system for high efficient regulation of brain homeostasis. Neuronal activity is known to regulate gap junction coupling through alteration of extracellular ions and neurotransmitters. However, the extent to which epileptic neuronal activity impairs the syncytial isopotentiality is unknown. Here, the neuronal epileptiform bursts were induced in acute hippocampal slices by removal of Mg^2+^ (Mg^2+^ free) from bath solution and inhibition of γ-aminobutyric acid A (GABA_A_) receptors by 100 µM picrotoxin (PTX). The change in syncytial coupling was monitored by using a K^+^ free-Na^+^-containing electrode solution ([Na^+^]_p_) in the electrophysiological recording where the substitution of intracellular K^+^ by Na^+^ ions dissipates the physiological membrane potential (V_M_) to ~0 mV in the recorded astrocyte. However, in a syncytial coupled astrocyte, the [Na^+^]_p_ induced V_M_ loss can be compensated by the coupled astrocytes to a quasi-physiological membrane potential of ~73 mV. After short-term exposure to this experimental epileptic condition, a significant closure of syncytial coupling was indicated by a shift of the quasi-physiological membrane potential to −60 mV, corresponding to a 90% reduction of syncytial coupling strength. Consequently, the closure of syncytial coupling significantly decreased the ability of the syncytium for spatial redistribution of K^+^ ions. Altogether, our results show that epileptiform neuronal discharges weaken the strength of syncytial coupling and that in turn impairs the capacity of a syncytium for spatial redistribution of K^+^ ions.

## 1. Introduction

Astrocytes establish the largest syncytium through gap junction coupling [1,2,3]. This anatomical attribute permits spatial buffering of K^+^ and Na^+^ ions and long-range redistribution of nutrients, metabolites and signaling molecules that are crucial for neuronal activity, tuning of neuronal oscillations, and brain energy metabolism [4,5,6,7,8]. The importance of this astrocytic network in brain function has been best illustrated in mice deficient in astrocytic gap junction channels connexin 43 (Cx43) and 30 (Cx30); this genetic manipulation resulted in a massively disturbed K^+^ and glutamate homeostasis, interrupted synaptic long-term potentiation (LTP), and impaired sensorimotor and spatial memory tasks [9,10,11,12]. 

The electrolyte-filled gap junctions also serve as electrical conductors that equalize the voltages among coupled cells. Indeed, a strong electrical coupling does confer an isopotentiality to astrocyte network across the brain [13,14,15]. In a demonstrated case, we showed that only under the syncytial isopotentiality, a sustained driving force can be maintained for high efficient K^+^ uptake. This physiological mechanism should also facilitate Na^+^-dependent uptake systems that are copiously expressed in astrocytes, such as glutamate, GABA and glycine transporters [16]. 

The pathological relationship between epileptic neuronal activity and astrocytic gap junction coupling remains a matter of debate. For example, the syncytial coupling is speculated to counteract the hyperactivity and synaptic transmission through facilitation of the uptake of neuronal released K^+^ and glutamate. This view is supported by a gene knockout study; deletion of astrocytic Cx43/Cx30 increased spontaneous epileptiform activity and decreased seizure thresholds [10,12]. However, excessive Ca^2+^-waves and other intracellular signals in an astrocytic syncytium may synchronize the activity of neurons within the network, and this synaptic transmission independent mechanism could facilitate seizures in an intact astrocyte syncytial network [17,18].

It is possible that in keeping up with the demand for neuronal hyperactivity, epileptic neuronal firing may up-regulate the strength of syncytial coupling and therefore the capacity of astrocyte homeostatic function. On the other hand, excessive neurotransmitter released from firing neurons could also elevate the intracellular Ca^2+^ ([Ca^2+^]_i_) to pathological levels in astrocytes [19,20], and the latter is known to disrupt gap junctions [21,22]. Nevertheless, which of the above scenarios occurs to the astrocyte network is yet to be determined. 

To answer this question, the impact of epileptiform discharges on the coupling strength of astrocyte syncytium is examined with our newly developed electrophysiological method [23]. We show that this experimental epileptic condition severely impairs the coupling strength of hippocampal astrocyte syncytium. 

## 2. Materials and Methods

### 2.1. Animals

All of the experimental procedures were performed in accordance with a protocol approved by the Animal Care and Use Committees of The Ohio State University. The wild type C57BL/6J and BAC-ALDH1L1-eGFP transgenic mice of both sexes at postnatal days (P) 21-28 were used in the present study [15,24,25]

### 2.2. Preparation of Acute Hippocampal Slices and Freshly Dissociated Astrocytes

Hippocampal slices were prepared from P21-25 mice as we have previously reported [26]. Briefly, in each preparation, a brain was rapidly removed from skull and placed into ice-cold oxygenated (95% O_2_/5% CO_2_) slice cutting artificial cerebrospinal fluid (aCSF) with reduced Ca^2+^ and increased Mg^2+^ (in mM: 125 NaCl, 3.5 KCl, 25 NaHCO_3_, 1.25 NaH_2_PO_4_, 0.1 CaCl_2_, 3 MgCl_2_ and 10 glucose). Coronal hippocampal slices (250 μm thickness) were cut at 4 °C with a Vibratome (Pelco 1500, Ted Pella, Inc., Redding, CA, USA) and transferred to the oxygenated standard aCSF (in mM: 125 NaCl, 25 NaHCO_3_, 1.25 NaH_2_PO_4_, 3.5 KCl, 2 CaCl_2_, 1 MgCl_2_ and 10 glucose, osmolality 295 ± 5 mOsm; pH 7.3–7.4). Brain slices were allowed to recover from preparation damage for at least 1 h at room temperature before recording. For sulforhodamine 101 (SR101) staining [27], the slices were transferred to a beaker with slice-holding basket containing 0.6 μM SR101 in aCSF, and were incubated at 34 °C for 30 min. Then, the slices were transferred back to standard aCSF at room temperature before recording. In the experiment for manipulating coupling strength, brain slices were pretreated with aCSF containing 100 µM meclofenamic acid (MFA) for one hour before recording and perfused with the same solution during recording. To induce epileptiform discharges, brain slices were perfused with Mg^2+^ free-PTX aCSF (in mM: 125 NaCl, 25 NaHCO_3_, 1.25 NaH_2_PO_4_, 3.5 KCl, 2 CaCl_2_, 10 glucose, and 100 µM picrotoxin (PTX), osmolality 295 ± 5 mOsm; pH 7.3–7.4).

To prepare freshly dissociated astrocytes, hippocampal brain slices were first incubated for 30 min in oxygenated aCSF supplemented with 0.6 μM SR101 at 34 °C. Then, the CA1 regions were dissected out and cut into ~1 mm^3^ pieces and transferred into a 1.5 mL Eppendorf tube containing oxygenated aCSF supplemented with 24 U/mL papain and 0.8 mg/mL L-cysteine for 7 min at RT. After papain digestion, the tissues were gently triturated 5–7 times into a cell suspension, which was then transferred into the recording chamber [28,29]. Although the cell suspensions contain tissue blocks varying in the number of astrocytes, only single dissociated astrocytes were used in this study. 

### 2.3. Electrophysiology

For brain slice recording, individual hippocampal slices were transferred to a recording chamber (RC-22, Warner Instruments, Holliston, MA, USA) mounted on a BX51WI microscope (Olympus, Olympus American, Inc., Melville, NY, USA) equipped with infrared differential interference (IR-DIC) and were perfused with oxygenated aCSF (2.5 mL/min) at room temperature. 

Recording pipettes were fabricated from borosilicate capillaries (1.5/0.86 mm outer/inner diameter, Warner Instruments) using a Flaming/Brown Micropipette Puller (Model P-87, Sutter Instrument, Novato, CA, USA). When filled with pipette solution noted below, the pipettes had the open tip resistance of 2-5 MΩ. The standard pipette solution contained (in mM): 140 KCl or K^+^-gluconate, 0.5 CaCl_2_, 1 MgCl_2_, 5 EDTA, 10 HEPES, 3 Mg-ATP and 0.3 Na_2_-GTP that was titrated with KOH to pH 7.25–7.27. The final osmolality was 280 mOsm. In K^+^ free-Na^+^ containing, or K^+^ free-NMDG-Cl containing pipette solutions, the 140 mM KCl or K^+^-gluconate in the standard pipette solution was substituted by 140 mM NaCl, or NMDG-Cl, respectively. 

Whole-cell patch clamp recordings were performed using a MultiClamp 700A amplifier and pClamp 9.2 software (Molecular Devices, Sunnyvale, CA, USA). A minimum of 2 GΩ seal resistance was required before rupturing the membrane into whole-cell configuration. The membrane potential (*V*_M_) was read either in “I = 0” mode or measured directly in current clamp mode without applying any holding currents. For neuronal recording, the access resistance (*R*_a_), membrane resistance (*R*_M_), and membrane capacitance (C_M_) were measured from "Membrane test" protocol built into the pClampex9.2. For quality control, a *R*_a_ < 20 MΩ is used to include neurons into final data analysis. For the low *R*_M_ astrocytes, only the membrane input resistance (*R*_in_) was measured by “Resistance test” protocol in pClampex 9.2 (pulse: −63 pA/600 ms), and any recordings with *R*_in_ > 50 MΩ were discarded [30]. All the experiments were conducted at room temperature (20 ± 2 °C). The liquid junction potential was compensated for before establishment of cell-attached mode and confirmed to be at ~0 mV after experiments by withdrawal of recording pipette. 

### 2.4. Imaging of Aldh1l1-eGFP Astrocytes and Analysis

Acute hippocampal slices were prepared from P21-25 BAC-ALDH1L1-eGFP transgenic mice according to the same procedure as for electrophysiological recording. Hippocampal slices obtained were first allowed to recover in aCSF for one hour. Then hippocampal slices were then randomly divided into control (in standard aCSF), and Mg^2+^ free-PTX groups. After incubation with Mg^2+^ free-PTX or aCSF for 4.0 ± 1.0 h (*n* = 8–9 hemisphere brain slices), the slices were quickly washed by aCSF, then were incubated in 4% paraformaldehyde (PFA) in phosphate-buffered solution (PBS) for 90 min at room temperature. Tissue sections were then mounted onto microscope slides and were coverslipped with Fluoromount G (SouthernBiotech, SouthernBiotech Company, Birmingham, AL, USA). Z stack images (approximately 20–30 µm in depth) were acquired using an SP8 confocal microscope (Leica, Leica Microsystems Inc., Buffalo Graves, IL, USA). Acquisition parameters were held constant for both control and Mg^2+^ free-PTX groups. 

### 2.5. Chemical Reagents

SR101 was purchased from Invitrogen (New York, NY, USA). All other used chemicals and salts were purchased from Sigma-Aldrich (St. Louis, MO, USA). Meclofenamic acid (MFA) was directly dissolved in aCSF before experiment. 100 mM picrotoxin (PTX) were dissolved in dimethyl sulfoxide (DMSO) and stored in a −20 °C freezer prior to use. The stock solutions were diluted to the final experimental concentration just before each experiment.

### 2.6. Data Analyses

Image data were analyzed using NIH ImageJ software (ImageJ 1.43m, National Institutes of Health, Bethesda, MD, USA) and LAS X software (LAS X Small_2.0.0, Leica Microsystems, Buffalo Grave, IL, USA). Other data were analyzed using Origin software (Origin 8.0, OriginLab, Northampton, MA, USA). Data are reported as mean ± SEM. Shapiro-Wilk test was carried out for normality and F-test was carried out for variance homogeneity. Mean differences between groups were detected using Student’s t-test. For data that did not meet the assumptions of normality and homogenous variances, the nonparametric Mann-Whitney U test was used instead. Significance level was set at *p* < 0.05.

## 3. Results

### 3.1. Induction of Neuronal Epileptiform Discharges in Hippocampal Slices

**Figure 1:**
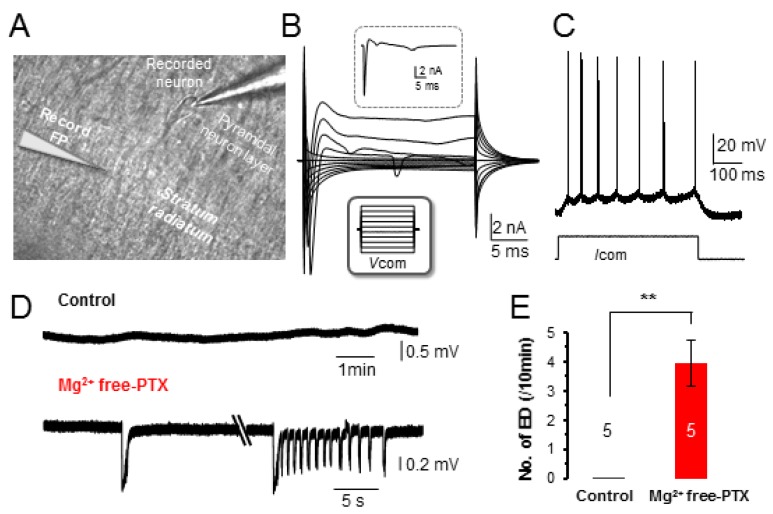

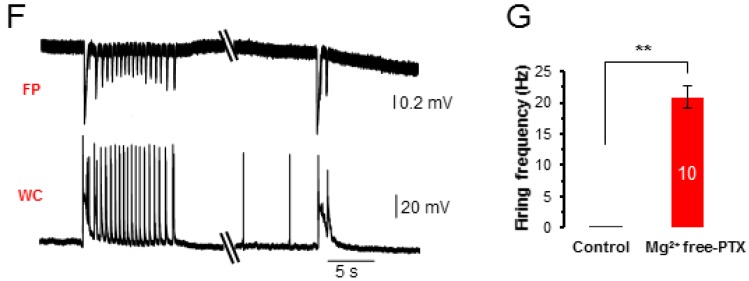
Epileptiform discharges induced by Mg^2+^ free-PTX. (**A**) Differential interference contrast (DIC) image of a portion of the CA1 region in an acute hippocampal slice. Pyramidal neurons could be readily identified from the pyramidal neuron layer based on cell morphology. Whole-cell (WC) recording from a pyramidal neuron first in the voltage-clamp (**B**)**,** then current-clamp (**C**). (**B**) In voltage-clamp recording, the pyramidal neuron was held at -70 mV at resting and then stepped to command voltages (*V*_COM_) from −160 mV to +20 mV at 20 mV increment and 25 ms duration (lower inset in **B**), and there was a 1 s interval between the consecutive *V*_COM_ steps. The pyramidal neuron was characterized by large depolarization step-activated inward Na^+^ and the following voltage-gated K^+^ channel currents; a 0 mV V_COM_-induced initial inward *I*_Na_ and following *I*_Ka_ and *I*_Kd_ are shown in the upper inset in (**B**,**C**) In the current-clamp recording, the current injection, 50 pA/ 500 ms induced multiple action potentials that fired with adaptation. (**D**) Field potential (FP) recording from *stratum radiatum* region (lower electrode in **A**), first in control, and then in Mg^2+^ free-PTX as indicated. Two different forms of epileptiform discharge (ED) are shown. (**E**) The frequency of ED events in Mg^2+^ free-PTX compared to control. (**F**) WC and FP dual recording; high frequency of neuronal firing is temporally phase-locked with ED. (**G**) High frequency of neuronal firing during ED events compared to non-ED events. **: *p* < 0.01.

To determine how excessive neuronal firing affects syncytial isopotentiality, a modified aCSF with removal of 1 mM MgCl_2_ and addition of 100 µM PTX (Mg^2+^ free-PTX solution) was bath perfused to hippocampal slices. To confirm that neuronal epileptiform discharges could be readily induced, membrane potential (*V*_M_) of hippocampal CA1 pyramidal neurons were recorded. Pyramidal neurons in CA1 region were identified based on distinct morphology and location (Figure 1A). These neurons showed a resting membrane potential (*V*_M_) of −61.2 ± 0.9 mV (*n* = 15 recordings from three mice), a membrane resistance (*R*_M_) of 154.1 ± 11.2 MΩ (n=10 recordings from three mice), and a membrane capacitance (C_M_) of 91.7 ± 8.9 pF (*n* =10 recordings from three mice). In voltage-clamp recording, the depolarization steps induced a sequential activation of voltage-gated inward Na^+^ (*I*N_a_), outward transient K^+^ channels (*I*K_a_) and delayed rectifying K^+^ channels (*I*K_d_) (Figure 1B). These properties were consistent with our previous reports [29,31,32].

In current-clamp recording, the CA1 pyramidal neurons were quiescent at resting conditions. The action potentials could only be induced by positive current injection, and the induced multiple spikes exhibited a characteristic adaptation (Figure 1C) [31,32]. Upon exposure to Mg^2+^ free-PTX solution, epileptiform discharges (EDs) at variable durations were induced (Figure 1D) at the frequency of 4.0 ± 0.8/min (*n* = 5 recordings from three mice, *p* < 0.01). To further correlate the ED events to the firing of individual neurons, neuronal whole-cell and field potential were simultaneously recorded (Figure 1A). Indeed, the onset of the burst firing of recorded neurons was always phase-locked with the ED events (Figure 1F). The burst neuronal firing reached to a frequency of 20.9 ± 1.8 Hz (*n* = 10 EDs from three mice, *p* < 0.01). Together, we showed that Mg^2+^ free-PTX treatment indeed induced ED events as a results of synchronized firing of neurons [33]. 3.2. Acute Exposure to Mg^2+^ Free-PTX Conditions does not Alter the Anatomy of Astrocyte Syncytium

**Figure 2:**
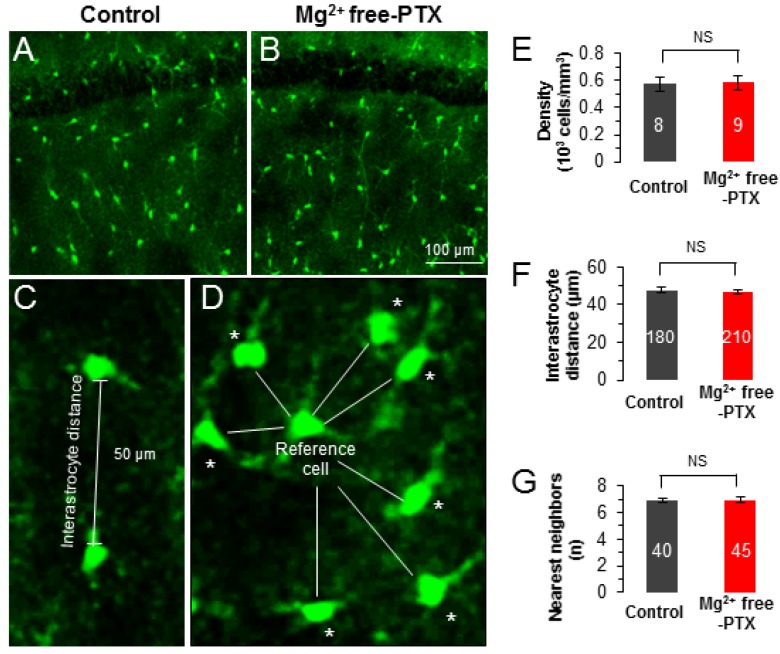
Acute exposure to Mg^2+^ free-PTX conditions does not alter the anatomy of astrocyte syncytium. (**A**,**B**) Confocal image of astrocyte networks in CA1 region from ALDH1L1-eGFP mouse of control group (**A**) and Mg^2+^ free-PTX group (**B**). The scale bar in (**B**) also applies to (**A**) (**C**,**D**) Representations of astrocyte syncytium anatomical parameters: interastrocyte distance and the nearest neighbors as indicated by asterisk (*). (**E**–**G**) Compared with the control group, no obvious differences in cell density (**E**), interastrocyte distance (**F**) and the number of nearest neighbors (**G**) were observed after treatment with Mg^2+^ free-PTX. The numbers inside the bar graphs indicate the hemisphere brain slices in (**E**), the counted pair of astrocytes in (**F**), the counted reference cells in (**G**), from **3** mice in each group. NS: *p* ≥ 0.05.

We have previously shown that each astrocyte is directly coupled to 7–9 of the nearest neighbors and this spatial arrangement is a prerequisite for syncytial isopotentiality to be achieved [13,14,15]. To answer if the Mg^2+^ free-PTX condition alters the cellular structure and spatial organization of astrocytes, we took advantage of BAC-ALDH1L1-eGFP transgenic mice to visualize astrocytes by their eGFP expression [14,15,25]. In acutely prepared brain slices, a feasible time window to examine the impact of the Mg^2+^ free-PTX conditions on astrocyte anatomy and function is around 2–6 hours. Therefore, the acute hippocampal slices were treated in oxygenated Mg^2+^ free-PTX solution for 4.0 ± 1.0 hours before confocal morphometric analysis (see Methods). At the cellular level, no obvious change in astrocyte morphology was found after treatment with Mg^2+^ free-PTX i.e., hypertrophy or shrinkage in cell body and processes. At the syncytial network level, astrocyte cell density remained unchanged (Figure 2A,B,E), so was the interastrocyte distance and the number of the nearest neighbors after Mg^2+^ free-PTX treatment (Figure 2C,D,F,G). Taken together, these results suggest that within the time window of Mg^2+^ free-PTX treatment, the anatomy of astrocytes remains intact.

### 3.2. Mg^2+^ Free-PTX Condition Depolarizes Astrocyte V_M_ without Altering of Passive Conductance

**Figure 3:**
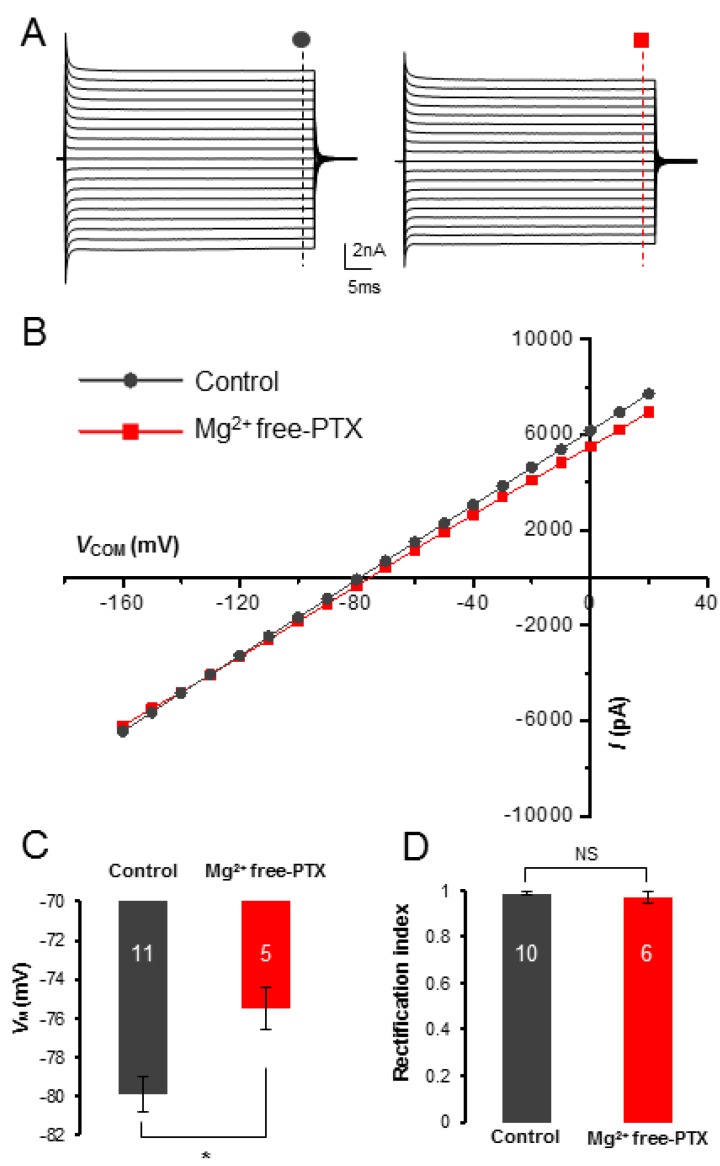
Epileptiform neuronal discharges do not induce changes in astrocyte passive conductance but depolarizes astrocytes. (**A**,**B**) The passive membrane conductance was not altered by Mg^2+^ free-PTX treatment, in terms of current amplitude and the linearity in current-voltage (I-*V*_M_) plot. (**C**) The Mg^2+^ free-PTX perfusion depolarized astrocyte *V*_M_ by 4.4 mV compared to control group in [K^+^]_p_ recording. (**D**) The rectification index (RI) values were comparable between Mg^2+^ free-PTX treated astrocytes and control group. *: *p* < 0.05, NS: *p* ≥ 0.05. In summary, the characteristic passive behavior of astrocyte membrane conductance remained unchanged after Mg^2+^ free-PTX treatment. Therefore, the observed *V*_M_ depolarization is likely a consequence of an elevated [K^+^]_e_ induced by epileptic neuronal firing, instead of change in the intrinsic property of astrocyte membrane K^+^ channels.

Neuronal firing elevates extracellular K^+^ concentration ([K^+^]_e_) [34,35,36]. After exposure to Mg^2+^ free-PTX solution, astrocyte *V*_M_ depolarized from −79.9 ± 0.9 mV (*n* = 11 recordings from 7 mice) to −75.5 ± 1.1 mV (*n* = 5 recordings from 3 mice, *p* < 0.05, Figure 3C). This is consistent with the notion that epileptiform neuronal activity elevates [K^+^]_e_. 

To determine whether Mg^2+^ free-PTX condition also affect the functional K^+^ channels that in turn contributed to the depolarized astrocyte *V*_M_, we used voltage-clamp recording to examine the current profile of astrocyte K^+^ channel expression. The passive behavior of astrocyte membrane conductance is known to reflect the intrinsic properties of known and unknown K^+^ channels, including inwardly rectifying K^+^ channel [28,30,37,38]. We found that the overall passive conductance was not altered by Mg^2+^ free-PTX treatment (Figure 3A,B). To further examine this, we compared the rectification index (RI) between control and Mg^2+^ free-PTX treatment groups [39]. The RI values were comparable between control (0.99 ± 0.01, *n* = 10 recordings from 10 mice) and Mg^2+^ free-PTX treatment group (0.97 ± 0.03, *n* = 6 recordings from 6 mice, *p* > 0.05, Figure 3D). 

### 3.3. Epileptiform Neuronal Discharges Impair the Strength of Syncytial Isopotentiality

**Figure 4:**
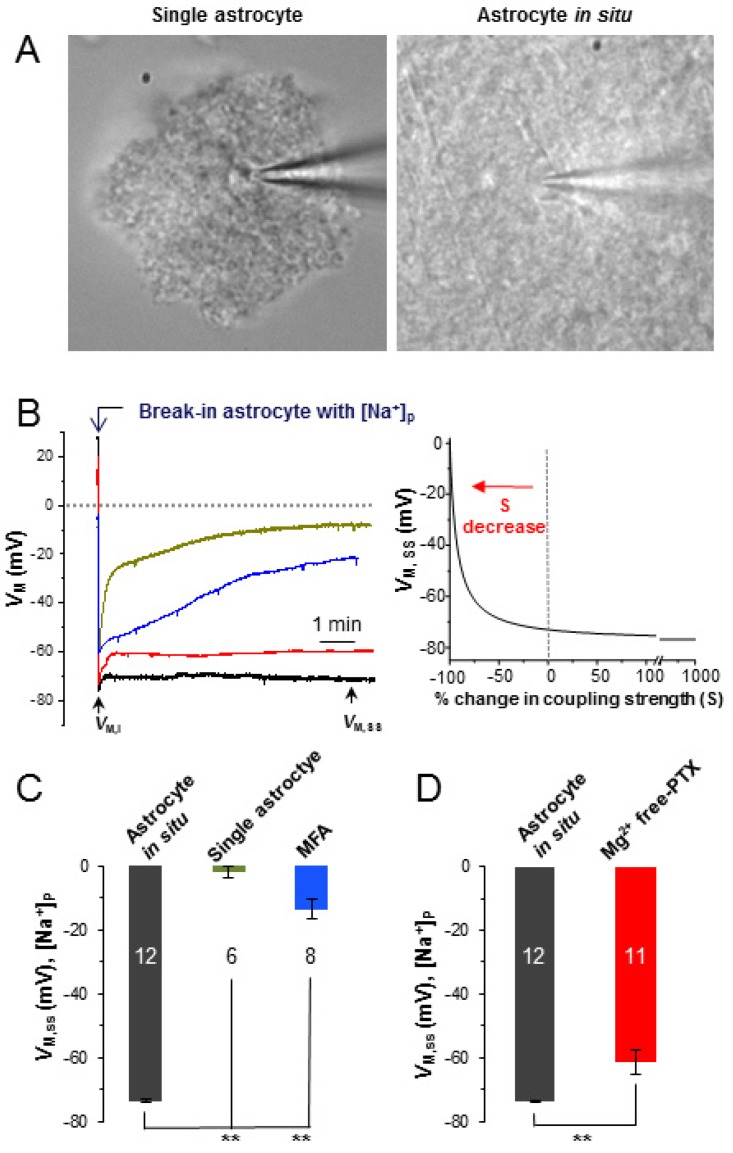
Epileptiform neuronal discharges impair the strength of syncytial coupling. (**A**) DIC images of a single freshly dissociated astrocyte (left), and an astrocyte in situ (right). (**B**) *V*_M_ recordings from an astrocyte in situ (black trace), a single astrocyte (green trace), an astrocyte in situ with MFA treatment (blue trace) and an astrocyte in situ with Mg^2+^ free-PTX treatment (red trace). On the right panel, a computational modeling illustrates an exponential relationship between the coupling strength (S) and the steady-state *V*_M_ (*V*_M,SS_) recorded from [Na^+^]_p_ recording. (**C**) Substitution of intracellular K^+^ content by [Na^+^]_p_ resulted in depolarization of *V*_M,SS_ to −1.8 ± 1.7 mV in a single astrocyte following Nernstian prediction. In the presence of MFA, the *V*_M,SS_ of an astrocyte in situ reached to a similar level as that of single dissociated astrocyte at −13.5 ± 3.1 mV. In syncytial coupled astrocytes, however, the *V*_M,SS_ remained at a quasi-physiological level of −73.7 ± 0.5 mV. (**D**) The Mg^2+^ free-PTX perfusion shifted the *V*_M,SS_ from control level of −73.7 ± 0.5 mV to −61.3 ± 3.7 mV. **: *p* < 0.01.

Substitution of intracellular K^+^ content by the Na^+^ ions should depolarize at the recorded astrocyte to ~ 0 mV following to the Nernstian prediction. This indeed occurs in the single freshly dissociated astrocyte (Figure 4A, left) where rupture of an astrocyte by [Na^+^]_p_ resulted in a progressive *V*_M_ depolarization to 0 mV (Figure 4B, green trace). Note that immediately after membrane rupture, the initial *V*_M_ (*V*_M_, _I_) reflects the resting *V*_M_ of the cell, whereas the *V*_M_ at the steady-state level (*V*_M,SS_) reflects the *V*_M_ of Nernstian prediction [13,15]. The *V*_M,SS_ is −1.8 ± 1.7 mV in freshly dissociated single astrocytes (*n* = 6 recordings from five mice) (Figure 4C, green bar).

In a syncytial coupled astrocyte (Figure 4A, right), however, the *V*_M,SS_ (−73.7 ± 0.5 mV, *n* = 12 recordings from five mice) remained at a quasi-physiological level due to compensation of “lost” *V*_M,SS_ by the coupled syncytium (Figure 4B, black trace; Figure 4C,D, black bar). To further confirm this, we used a gap junction inhibitor, 100 µM MFA, to block syncytial coupling [3,40]. 

Our previous study showed that 100 µM MFA eliminates gap junction coupling by 99.3% [13]. In the presence of MFA, the *V*_M,SS_ (−13.5 ± 3.1 mV, *n* = 8 recordings from six mice), to a great extent recaptured the *V*_M,SS_ level showed in single isolated astrocytes (Figure 4B, blue trace, Figure 4C, blue bar). 

After Mg^2+^ free-PTX perfusion, the *V*_M,SS_ positively shifted to −61.3 ± 3.7 (*n* = 11 recordings from eight mice, Figure 4B, red trace; Figure 4D, red bar) compared to the control group of −73.7 ± 0.5 mV (*n* = 12) (*p* < 0.01). According to our computational model prediction, this positive shift of *V*_M,SS_ corresponds to a near-complete inhibition of the strength of syncytial coupling at 90% [15]. However, in [K^+^]_P_ recording, Mg^2+^ free-PTX treatment also depolarized astrocytes by 4.4 mV (Figure 3C), mostly caused by neuronal firing induced high [K^+^]_e_. Taking this into consideration in our computational modeling, the Mg^2+^ free-PTX induced inhibition of syncytial coupling is estimated to be 84%. Therefore, the epileptiform discharges severely impairs the strength of astrocyte syncytial isopotentiality.

### 3.4. Epileptiform Discharges in Slices Impair the K^+^ Redistribution Capacity of an Astrocyte Syncytium

**Figure 5:**
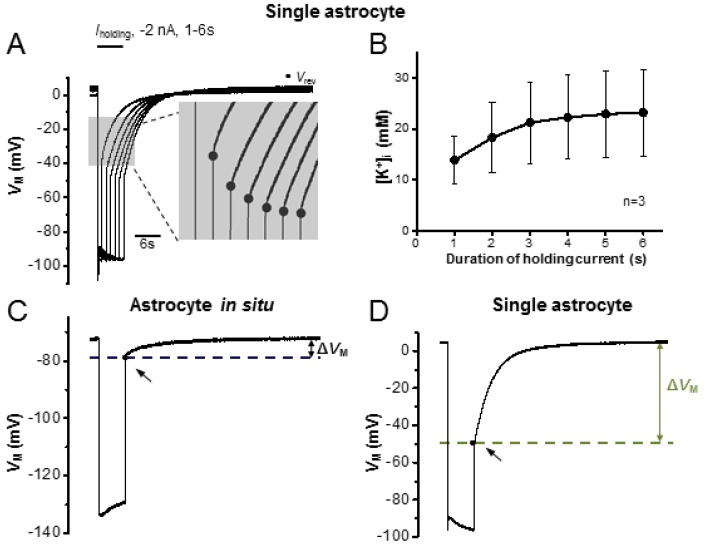

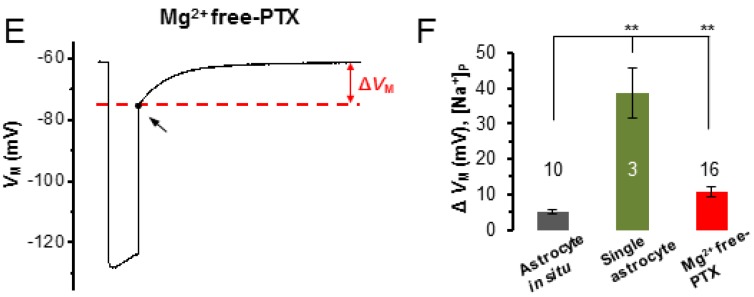
Epileptiform neuronal discharges impair the capacity of a syncytium for K^+^ redistribution. (**A**) Single astrocyte recorded with a [Na^+^]_p_ in current-clamp mode; −2 nA current steps (*I*_holding_) were applied at incremental durations from 1 to 6 s. In between these steps, the cell was maintained at resting condition for *V*_M_ recovery. The longer the duration of the current steps, the stronger the negative shift in the reversal potential (*V*_rev_) upon withdrawal of the steps, indicating more accumulation of K^+^ inside astrocytes. (**B**) Based on the *V*_rev_ values recorded in single astrocytes, the [K^+^]_i_ values were calculated according to the Goldman-Hodgkin-Katz equation, and are plotted against the step pulse durations. (**C**–**E**) In [Na^+^]_p_ recording, the Δ*V*_M_ is the difference between the basal *V*_M_ and *V*_rev_ values, and is used to compare the capacity of K^+^ redistribution under indicated conditions. Representative recordings of Δ*V*_M_ from an astrocyte in situ (**C**), a single astrocyte (**D**) and an astrocyte with Mg^2+^ free-PTX treatment (**E**). (**F**) Comparison of Δ*V*_M_ between astrocytes in situ with single astrocytes, and with astrocytes after treating with Mg^2+^ free-PTX, a significant change occurred to both comparisons, indicating Mg^2+^ free-PTX treatment significantly impairs the redistribution capacity of an astrocyte syncytium. **: *p* < 0.01.

The clearance of K^+^ released from active neurons is a major function of astrocytes. In the K^+^ spatial buffering hypothesis, K^+^ ions uptaken from a local area need to be spatially transferred across gap junctions and, in turn, be released to regions with less neuronal activity [41,42]. Therefore, a strong K^+^ redistribution capacity is required. A severely reduced syncytial coupling implies an impaired spatial redistribution capacity for K^+^ due to the insult of Mg^2+^ free-PTX treatment. To test this, we used [Na^+^]_p_ to create a “K^+^-deficient astrocyte” as a “reporter” for the increase of [K^+^]_i_ in the recorded cell. As shown in Figure 5A,B, from a single isolated astrocyte an uptake driving force is created by using of −2 nA current steps at varied duration from 1–6 s. A step duration-dependent increase in [K^+^]_i_ is indicated by the negative shift in *V*_rev_ values at the end of the steps, and the net accumulation of [K^+^]_i_ can be calculated from these *V*_rev_ values according to Goldman-Hodgkin-Katz (GHK) equation [25,28]. In the GHK equation calculation, the *P*_Na_/*P*_K_ and *P*_Cl_/*P*_K_ and *P*_Ca_/*P*_K_ were assumed at 0.015, 0.0 and 0.0, respectively [5,26]. As the step duration increased from 1 to 6 seconds, there was an incremental increase in [K^+^]_i_: 13.9 ± 4.7 mM (1 s), 18.3 ± 6.9 mM (2 s), 21.2 ± 8.0 mM (3 s), 22.3 ± 8.2 mM (4 s), 23.0 ± 8.5 mM (5 s) and 23.2 ± 8.5 mM (6 s) (*n* = 3 recordings from three mice, Figure 5B). 

In a syncytial coupled astrocyte, the *V*_rev_ is not only affected by the negative holding current but also altered by syncytial isopotentiality. Therefore, only the relative change in *V*_rev_ (Δ*V*_M_) can be used for comparison of K^+^ redistribution capacity, while the intracellular K^+^ contents calculation is not feasible in this condition. As shown in Figure 5C, in syncytial coupled astrocytes, only a minor Δ*V*_M_ occurred in response to a 6 s step, 5.08 ± 0.45 mV (*n* = 10 recordings from three mice). In the group treated with Mg^2+^ free-PTX, the inhibition of syncytial coupling is indicated by a larger Δ*V*_M_, 10.74 ± 1.55 mV (*n* = 16 recordings from seven mice, Figure 5E), compared to astrocyte in situ (*p* < 0.01, Figure 5F). For comparison, the largest Δ*V*_M_ was induced from single isolated astrocytes, 38.62 ± 7.02 mV (*n* = 3 recordings from three mice, Figure 5D). Altogether, we show that the Mg^2+^ free-PTX treatment severely impairs the K^+^ redistribution capacity in astrocyte syncytium.

## 4. Discussion

Despite decades of investigation, the impact of seizure activity on astrocyte gap junction coupling remains controversial. A seizure-induced increase, decrease or unaltered expression of gap junction channels, connexin 43 and 30, have all been reported at both the transcript and protein levels in human epilepsy and in animal epilepsy models [43,44]. 

At the functional levels, the results were also contradictory. In a genetic mouse model of tuberous sclerosis complex, a human disease associated with medically intractable seizures, a significant reduction of inter-astrocytic dye coupling has been reported in the hippocampal CA1 region [45]. On the contrary, an increase in astrocyte dye coupling has been reported in a rat model of temporal lobe epilepsy (systemic kainate injection) during the latent 7–16 days post status epilepticus [46]. An increased coupling has also been found in fluorescence recovery measurement after photobleaching (FRAP) in hippocampal slice cultures after chronic exposure to GABA_A_ receptor inhibitor bicuculline, which developed a chronic in vitro model of epilepsy [47]. 

### 4.1. Epileptiform Neuronal Discharges Impair Astrocyte Gap Junction Coupling

To address this controversial and important question, a common Mg^2+^ free-PTX model was used to induce epileptiform discharges in hippocampal slices. In the present study, we intended to examine the acute effect of Mg^2+^ free-PTX treatment on the anatomy and function of the astrocyte syncytium. The epileptiform discharges could be readily induced (Figure 1). Importantly, within this relatively short time window, neither the cellular morphology nor the spatial organization pattern of astrocytes was noticeably altered. Therefore, this model allowed us to examine the acute impact of epileptiform discharges on astrocyte syncytial coupling strength. 

The acute functional impact of epileptiform discharges on syncytial isopotentiality has been explored with a new electrophysiological method recently developed by us [23]. Using this method, we show that epileptic neuronal activity weakens the syncytial coupling; this is indicated by a positive shift of the steady-state quasi-physiological *V*_M_ (*V*_M,SS_) (Figure 4). A 12.4 mV positive shift in *V*_M,SS_ corresponds to a 90% inhibition of astrocyte syncytial coupling [15]. 

The basal level of astrocyte *V*_M_ fluctuates minimally following the change of [K^+^]_e_ in vivo [48,49,50]. Intensive neuronal activity, however, can increase [K^+^]_e_ to a ceiling level of 12 mM in active neuronal zones. In our studies, Mg^2+^ free-PTX treatment induced a 4.4 mV *V*_M_ depolarization, whereas the passive conductance remained intact (Figure 3). These results indicate that elevation of [K^+^]_e_ should be mostly accountable for Mg^2+^ free-PTX treatment induced astrocyte *V*_M_ depolarization. 

It should be noted that in [Na^+^]_p_ recording, part of the positive shift of *V*_M,SS_ (12.4 mV) could also be attributable to [K^+^]_e_ increase. Nevertheless, after correction of this portion of *V*_M_ depolarization, the Mg^2+^ free-PTX treatment induced syncytial coupling inhibition still reaches ~84% based on our model prediction [15,23].

### 4.2. Epileptiform Discharges Impair the Redistribution Capacity of a Syncytium for K^+^ Ions

The weakening of syncytial coupling should affect the redistribution capacity of a syncytium for K^+^ ions. Assuming K^+^ spatial buffering does serve as a critical homeostatic mechanism, a significant closure of network coupling should severely affect the K^+^ homeostasis in the brain. In the present study, this possibility has been tested by comparison of the K^+^ redistribution capacity from a syncytial coupled astrocyte after the experimental accumulation of intracellular K^+^ ions. We found that indeed weakened syncytial coupling significantly attenuated the ability of astrocytes to redistribute experimentally accumulated K^+^ ions to the coupled syncytium after treating the slices with Mg^2+^ free-PTX. 

### 4.3. Acute vs Chronic Impact of Epileptiform Discharges on Astrocyte Syncytium

Epilepsy is often accompanied by massive reactive gliosis in patients [51]. This observation has also been simulated in animal models of epilepsy [52]. Astrogliosis disrupts the mutually exclusive astrocytic domains that lead to an extensive interdigitation of astrocyte processes. It has been speculated that this pathological change may enhance astrocyte-astrocyte contact and hence a stronger coupling [46]. However, another report has shown the lack of dye coupling among proliferating reactive astrocytes [53]. 

In the present study, we asked if epileptiform discharges could exert acute alterations to the structure of individual astrocytes as well as their established syncytium. Our results show that after short-term exposure to epileptic insults, no obvious change was induced in astrocyte morphology in terms of hypertrophy. Also, astrocyte cell density and the pattern of spatial organization were not altered by Mg^2+^ free-PTX treatment. These results indicate that the functional state of a syncytium could be altered way ahead of the pathological manifestation in the morphology of individual astrocyte and astrocyte syncytium. 

Although the underlying mechanisms are yet to be identified, several regulatory mechanisms of gap junction coupling and syncytial isopotentiality could be initiated by neuronal activity. First, the control ability of syncytial isopotentiality weakens with expanding high K^+^ affected areas inside a syncytium. Second, the coupling strength (*s*) weakens when membrane K^+^ conductance (G_K_) increases under a constant gap junction conductance (G_g_) (*s* = G_g_/G_K_) [13]. In regions experiencing intensive neuronal activity, a high K^+^ influx rate in astrocytes, or increased G_K_, is inevitable as prediction by Goldman-Hodgkin-Katz (GHK) current equation [54]. This can, in turn, lead to a weakening coupling strength. Thus, disruption of syncytial isopotentiality is expected during intensive neuronal activity. Third, it is well-established that glutamatergic signaling induces intracellular Ca^2+^ elevation in astrocytes [55,56,57]. A previous study showed that 50 nM–500 nM [Ca^2+^]_i_ dose-dependently increases the open probability of Cx43, whereas further increase in [Ca^2+^]_i_ from 500 nM to 1 mM progressively inhibits Cx43 [58]. Because of the complexity, further experiments are necessary to uncover in detail the dynamic interactive mechanism between a syncytium and its associated neuronal networks. 

The role of astrocytes in epilepsy has attracted increasing research attention, therefore it is possible that the future anti-epileptic therapies may target specifically on astrocytes. In the present study, our study provides the first evidence that the dysfunction of syncytium indeed contributes to the pathology of epilepsy; therefore, gap junction coupling could be a promising target for the development of future antiepileptic treatment.

## 5. Conclusions

Our anatomical and electrophysiological studies show that despite no change in the gross anatomy of astrocyte syncytium, a short-term bout of epileptiform neuronal discharge is sufficient to weaken the strength of syncytial coupling and impair the capacity of a syncytium for spatial redistribution of K^+^ ions.
